# Mindfulness Meditation Biases Visual Temporal Order Discrimination but Not Under Conditions of Temporal Ventriloquism

**DOI:** 10.3389/fpsyg.2020.01937

**Published:** 2020-08-07

**Authors:** Yue Tian, Xinghua Liu, Lihan Chen

**Affiliations:** ^1^School of Psychological and Cognitive Science, Peking University, Beijing, China; ^2^Beijing Key Laboratory of Behavior and Mental Health, Peking University, Beijing, China

**Keywords:** mindfulness meditation, temporal ventriloquism, audiovisual, temporal order judgment, attention

## Abstract

This study examined how cognitive plasticity acquired from a long (8 weeks) course of mindfulness training can modulate the perceptual processing of temporal order judgment (TOJ) on a sub-second scale. Observers carried out a TOJ on two visual disks, with or without concurrent paired beeps. A temporal ventriloquism paradigm was used in which the sound beeps either were synchronized with the two disks or bracketed the visual stimuli by leading the first disk by 50 ms and lagging the other by 50 ms. A left-to-right bias in TOJ was found under the visual-only condition after mindfulness training. This bias was positively correlated with “acting with awareness,” a factor in the Five Facet Mindfulness Questionnaire, showing that awareness of every moment and enhanced attention focus magnify the left-to-right bias. However, the effect of mindfulness training may be short-lived and was not present when attention was diverted by auditory events in the cross-modal temporal ventriloquism illusion.

## Introduction

Mindfulness has been defined by Kabat-Zinn as “paying attention in a particular way, on purpose, in the present moment, and non-judgmentally” (Kabat-Zinn, 1990). Recently, mindfulness has become a popular and effective psychological intervention (Van Dam et al., 2018; Choo et al., 2019; Hadash and Bernstein, 2019; Raffone et al., 2019). Mindfulness-based interventions (MBIs) such as the Mindfulness-Based Stress Reduction Program (MBSR) (Kabat-Zinn, 1990) and Mindfulness-Based Cognitive Therapy (MBCT) (Teasdale et al., 2000) alleviate psychological symptoms (e.g., anxiety and mood symptoms) with medium effect sizes (Hofmann et al., 2010). Through such training, high-level cognitive functions including meta-cognitive awareness and attention can be improved through enhanced working memory capacity (Davis and Hayes, 2011). Mindfulness meditation can change practitioners’ moment-to-moment time perception by helping them pay more attention to the immediate experience of the “here and now” (i.e., the “subjective present”) (Elliott et al., 2006; Arstila and Lloyd, 2014; Elliott and Giersch, 2015; Wang et al., 2015; Murakami, 2017; Elliott, 2019).

Mindfulness training has generally been found to cause a temporal dilation effect in a bisection task, resulting in an overestimation of the duration of a target stimulus (Kramer et al., 2013; Droit-Volet et al., 2015; Singh and Srinivasan, 2019). In these studies, observers compared probe durations with standard “short” (such as 400 ms) and “long” (such as 1,600 ms) references, after short (within a few hours) or longer (several weeks) courses of training in mindfulness meditation. The sensitivity of timing was dependent on the length of the training period. Long-term mindfulness training increased participants’ attention to time perception in a way that led to overestimation, while one-shot exercise did not (Droit-Volet et al., 2015).

Time perception on sub-second and second timescales mobilizes different mechanisms: perceptual processes at the sub-second timescale and cognitive processes at the second timescale (Rammsayer and Lima, 1991). Previous studies involving bisection tasks employed a wide range of time durations, which makes it difficult to differentiate the perceptual vs. cognitive processes that came into play when participants performed the timing task (Kramer et al., 2013; Droit-Volet et al., 2015; Singh and Srinivasan, 2019). Temporal order judgment (TOJ) is an important form of time perception that is attention-demanding and should be a good probe for investigation of timing perception. Visual stimuli presented at the location where attentional capture happened by auditory cueing will be perceived to have been presented earlier than those that have not received an auditory cue, and the accuracy of TOJ can be improved by cross-modal exogenous orienting (Santangelo and Spence, 2008, 2009). However, TOJ has seldom been used to probe the effects of mindfulness training. The present study used a TOJ task in which the time range (with respect to the total duration including the stimuli and the gap in between) was below 500 ms, and observers thus interpreted stimuli mainly through perceptual processing rather than through cognitive engagement (Rammsayer and Lima, 1991; Lewis and Miall, 2003b).

Specifically, this study examined whether long-term mindfulness training would have an impact on the perceived temporal order of stimuli. As a means to investigate both the constraints of the training effect and the role of selective attention, a temporal ventriloquism task was used in which the subjectively perceived time onsets of visual stimuli were “pulled” away by task-irrelevant, temporally asynchronous (with respect to the onsets of visual stimuli) sound beeps that bracketed paired visual stimuli (Fendrich and Corballis, 2001; Morein-Zamir et al., 2003; Shi et al., 2010; Chen and Vroomen, 2013). We expected that long-term mindfulness training would enhance the participants’ attentional focus on the two visual stimuli (“markers”) that enclosed the gap interval and thereby lead to a time expansion effect upon the two markers (Brown, 1985; Tse et al., 2004; Kanai and Watanabe, 2006). Since the general attentional capacity would not be changed (Trautwein et al., 2020), the “gap” interval between the two visual stimuli might be compressed, and discrimination of the TOJ would thus be hampered. On the other hand, if the training effect were short-lived, the accompanying beeps (one leading the onset of the first visual stimulus and the other lagging the second) might cause the above effect in unisensory (visual) attention to be diminished or even abolished due to the diverting of focus from visual to auditory attention by the temporal ventriloquism effect. Therefore, it was hypothesized that the just-noticeable difference (JND) of the TOJ task would decrease after long-term mindfulness training under visual-only conditions but might not change under conditions that include a temporal ventriloquism effect.

The point of subjective equality (PSE) is another common index of TOJ tasks. It is expected that a left-to-right bias resulting from slight shifts of the PSEs will be enhanced after long-term mindfulness training that improves participants’ attentional orienting (Grossman et al., 2004; Jha et al., 2007; Semple, 2010). Left-to-right bias may simply occur whenever participants apply their habit of scanning events according to the usual reading direction; this bias has been found in many other contexts (Chokron et al., 1997; Verleger et al., 2010; Karim et al., 2016; Ransley et al., 2018). Typically, during space-mapping, our attention tends to focus initially on the left and then moves to the right. Healthy individuals favor the left side of space: for example, they will be biased to bisect a horizontal line to the left of the veridical center (Jewell and McCourt, 2000; Nicholls and Loftus, 2007; Loftus et al., 2009). This left-to-right bias is also related to attentional orienting (Perez et al., 2009). It was predicted that after the mindfulness training, participants’ attentional orienting would be improved, and a larger shift of the PSEs might be observed under the visual-only TOJ task. However, this shift of the PSEs may not be observed under conditions of temporal ventriloquism, because auditory stimuli tend to capture one’s attention automatically (Koelewijn et al., 2009).

## Materials and Methods

### Participants

The required sample size for a statistical test of within–between interaction was calculated in advance using G^∗^Power 3.1.9.4 (setting f = 0.25, 1−β = 0.9, α = 0.05) (Faul et al., 2009). The result showed that at least 24 participants were needed, with each group (experimental group and control group) having at least 12 participants.

Participants (age 24–53 years, mean 34.2 years old) without any mindfulness-related experience were recruited in Beijing and randomly assigned to the training group or the control group. A total of 25 complete data sets were collected (see below for details). The difference in age between the training group (*M* = 34.4, *SD* = 9.6) and the control group (*M* = 34.1, *SD* = 7.8) was not significant [*t*(23) = −0.097, *p* = 0.92]. All participants had normal or corrected-to-normal vision and gave written informed consent. The study was approved by the Academic Affairs Committee of the School of Psychological and Cognitive Sciences at Peking University.

### Stimuli and Apparatus

The center of the black disk was aligned with the horizontal centerline of the screen [R:192 B:192 G:192], and the refresh rate of the monitor was 100 Hz. The two disks were symmetrically positioned on the left and right visual fields. The radius of the disk was 0.8 degrees, and the distance between the center of the disk and the center of the screen was 3 degrees. The beeps used in the experiment had a frequency of 800 Hz and an amplitude of 60 dB, each lasting for 30 ms, with 5 ms of fade-in and fade-out.

Display of the stimuli was controlled by programs written in Matlab (Mathworks Inc.) and the Psychophysics Toolbox (Brainard, 1997; Pelli, 1997; Kleiner et al., 2007).

### Design and Procedure

**FIGURE 1:**
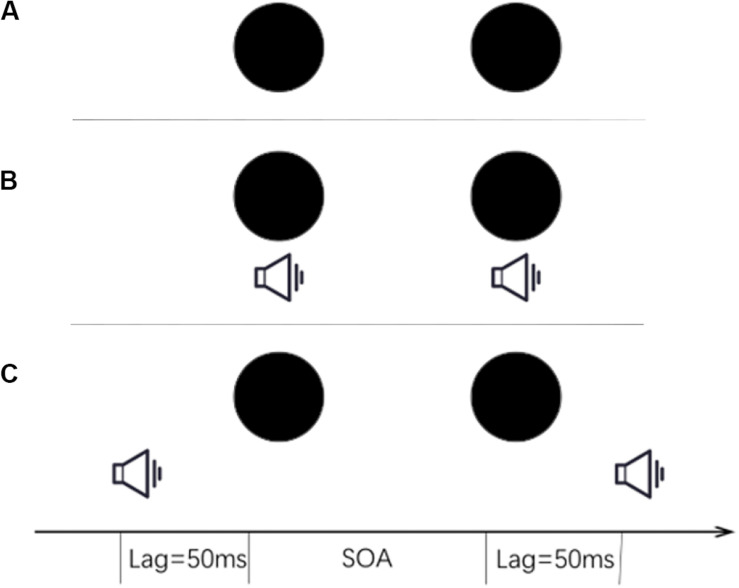
Audiovisual configurations. **(A)** Visual-only stimuli (no beep). **(B)** Two disks with two synchronized beeps. **(C)** Two disks with a leading beep (50 ms ahead of the first disk) and a lagging beep (50 ms behind the second disk). There were seven levels of stimulus onset asynchrony (SOA): −150, −100, −50, 0, 50, 100, and 150 ms (minus sign indicates that the right disk appeared first). The disks randomly appeared on the left or right side, separated by the above SOAs.

**FIGURE 2:**
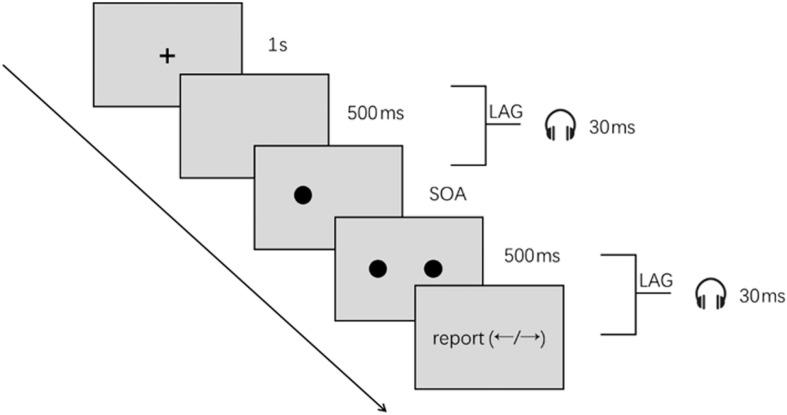
Stimuli configuration and task procedure. A black fixation point was first displayed for 1 s in the center of the screen with a gray background. The fixation point then disappeared, and 500 ms later, the disks were successively shown and beeps presented according to the given settings of the current trial. The second disk was presented for 500 ms, and the two disks disappeared at the same time. The participants pressed the left or right key of the keyboard to report whether they thought the first disk was presented at the left or right side of the screen.

The test consisted of two blocks. One of the blocks was conducted under visual-only conditions in which no beep was presented (Figure 1A). In the other block, the test was conducted under audiovisual conditions in which the beeps were presented along with the disks (Figures 1B,C). There were a total of 98 trials for the visual-only block and 196 trials for the audiovisual block in which trials of two different beep settings (Figures 1B,C) were randomly intermingled. Block order was balanced across participants. The order of the left and right disks was determined pseudo-randomly, and the time interval between the two disks could be −150, −100, −50, 0, 50, 100, or 150 ms (a minus sign indicates that the right disk appeared first) (Figure 2). Participants were instructed that if they judged that the left disk appeared first, they were to report it by pressing the left-arrow key of the keyboard. If they judged that the right disk appeared first, they were to press the right-arrow key to report.

Participants participated in two sessions of tests. The experimental group received a session in mindfulness training between the two sessions, whereas the control group did not. In the pre-training test, participants first filled out the Chinese revised version (Deng et al., 2011) of the Five Facet Mindfulness Questionnaire (FFMQ; Baer et al., 2006) on the computer and then completed the two blocks of TOJ tasks (i.e., visual and audiovisual), which took approximately 25 min. Participants wore headphones during the tasks and were required to complete a practice section before the formal data collection tests. Participants were then randomly assigned to either the 8-week mindfulness training group (12 participants, 4 males) or the wait-list control group (13 participants, 2 males). Eight weeks later, both groups received a post-training test with the same procedure as the pre-training test. As a reward, a 2-day intensive mindfulness training workshop was administered to the control group immediately after the post-training test.

### Mindfulness Training

The benefits of mindfulness on mental health and emotion management were explained to participants, but any effect on cognitive ability was not mentioned. The mindfulness training was designed based on the protocol for mindfulness-based cognitive therapy (MBCT) (Segal et al., 2002) and mindfulness-based stress reduction (MBSR) (Kabat-Zinn, 1990), emphasizing a focus on the present and non-judgmental awareness. Since the participants were not clinical clients, depression-related content was replaced with other meditation practices. The training consisted of four parts: (1) body scans (paying attention to the sensation of the body from head to toe); (2) sitting meditation (focusing on and experiencing one’s breath or thoughts while sitting comfortably); (3) walking meditation (observing and experiencing the sensation of body parts’ movement without judgment); and (4) mindfulness yoga (focusing on and maintaining stretching). This group training took 2.5 h each week and lasted for a total of 8 weeks. In addition to the weekly training, at least 30 min of home mindfulness practice was required, and participants were asked to record their daily practice and impressions. During group discussion, participants could share their feelings and receive any necessary guidance. The instructors of the mindfulness training had over 4 years of personal experience in mindfulness and more than 2 years of group-teaching experience. They were not informed of the purpose of the study and the training.

### Five Facet Mindfulness Questionnaire (FFMQ)

The self-report measure of the FFMQ consisted of 39 items assessing five factors, which could be classified into the following subscales: *observing* (noticing and being aware of internal and external stimuli, including sensation, emotion, cognition, and perception), *describing* (describing internal experiences with words either verbally or mentally), *acting with awareness* (focusing on one’s current activities and consciously paying attention to each experience), *non-judging* (accepting all experiences of the current moment), and *non-reacting* (not making a habitual automatic response). Items were scored on a five-point Likert-type scale (1 = never or very rarely true, 5 = very often or always true; some items were reverse-scored). Higher scores indicated a more advanced level of mindfulness. In this study, we adopted the Chinese version of the FFMQ, which has been tested and found to have acceptable psychometric properties and is thus a valid instrument for the assessment of mindfulness (Deng et al., 2011).

## Results

**FIGURE 3:**
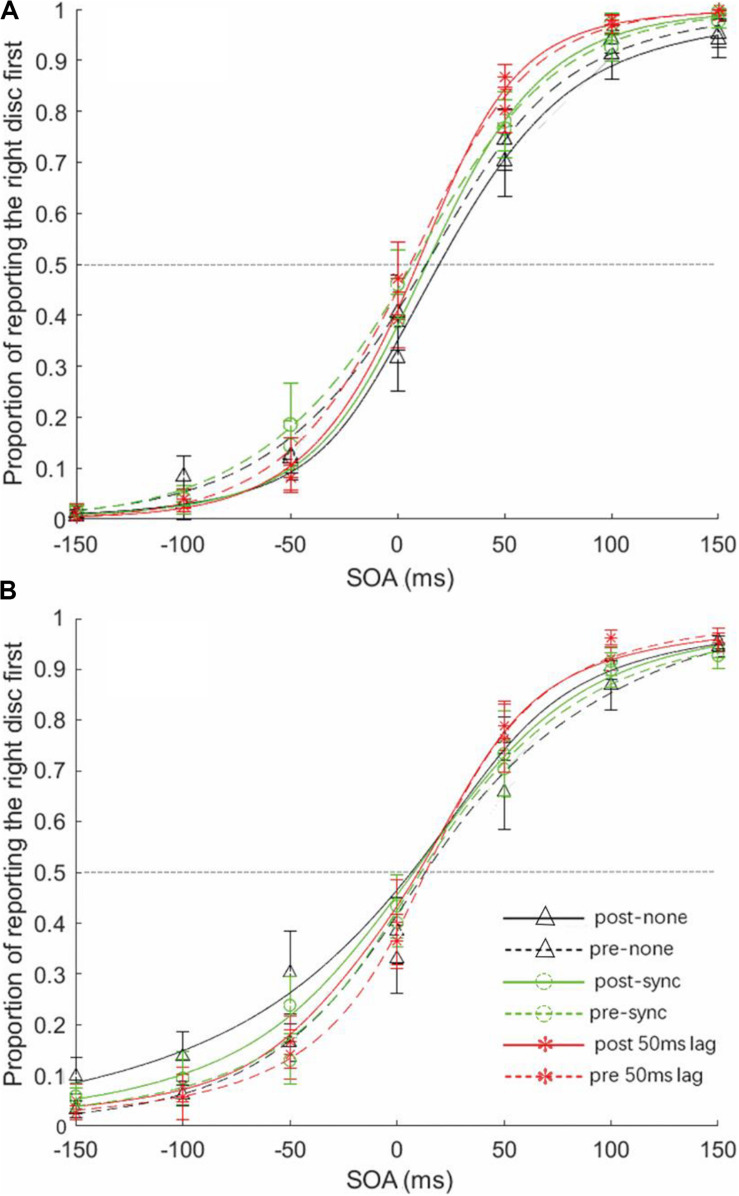
Averaged psychometric curves. **(A)** The psychometric curves for the pre- and post-training test in the mindfulness-trained group. **(B)** The psychometric curves for the “pre” and “post” results in the control group. For both graphs, the solid lines represent post-test results, and the dotted lines represent pre-test results. The black lines represent the purely visual (no beeps) condition, the green lines represent the synchronous beeps condition, and the red lines represent the 50 ms lag beep condition. Error bars represent standard error.

Psychometric functions were calculated for each participant under each condition by fitting a cumulative Gaussian function to the percentage of the “right disk first” responses for the stimulus onset asynchronies (SOAs) (Figure 3). The JNDs were then calculated from each psychometric function by subtracting the value of the SOA at which 75% of “right first” responses were made from the SOA at which participants made 25% “right first” responses, and then dividing the difference by two. The PSE, indicating the SOA at which the participants were maximally uncertain concerning the temporal order of the stimuli, was also calculated from each function as the SOA at which 50% “right first” responses were made (Treutwein and Strasburger, 1999; Wichmann and Hill, 2001).

The mean PSE and JND of the TOJ task for both groups are listed in Tables 1, 2. The effect of mindfulness training was examined by first analyzing the data from the pre-training test (both JNDs and PSEs). The results revealed that the two groups had equal initial timing abilities in terms of PSEs [*F*(1, 23) = 0.542, *p* = 0.469, η^2^ = 0.023] and JNDs [*F*(1, 23) = 2.429, *p* = 0.133, η^2^ = 0.096]. This indicates that any effect found in this study was not due to the arrangement of the participants in different groups or potential initial biases of TOJ. To examine the effect of the 8-week mindfulness training on the TOJ task and the temporal ventriloquism effect, repeated measure ANOVA was implemented with a between-group factor of groups (training vs. control) and within-group factors of test sessions (pre vs. post) and beep settings (no beep vs. synchronized vs. 50 ms lag) on both PSE and JND data.

**TABLE 1:** Mean points of subjective equality (PSEs) with associated standard errors for each sound condition, both pre- and post-test (none: visual-only condition; 0 ms: audiovisual synchronous condition; 50 ms: audiovisual asynchrony with SOA of 50 ms).

		None	0 ms	50 ms
Training	Pre	12.36 (10.36)	5.10 (10.67)	4.64 (8.07)
Group	Post	25.80 (11.23)	9.30 (7.35)	7.70 (4.79)
Control	Pre	24.49 (8.80)	14.74 (9.01)	8.24 (6.89)
Group	Post	−5.26(11.27)	5.31 (9.47)	6.98 (7.61)

**TABLE 2:** Mean just-noticeable differences (JNDs) with associated standard errors for each sound condition, both pre- and post-test (none: visual-only condition; 0 ms: audiovisual synchronous condition; 50 ms: audiovisual asynchrony with SOA of 50 ms).

		None	0 ms	50 ms
Training	Pre	35.92 (3.56)	32.73 (2.57)	26.98 (2.20)
Group	Post	33.61 (5.09)	31.46 (2.71)	26.21 (2.25)
Control	Pre	43.75 (5.72)	49.73 (8.43)	35.29 (7.49)
Group	Post	54.98 (9.82)	49.52 (8.94)	42.71 (8.96)

**FIGURE 4:**
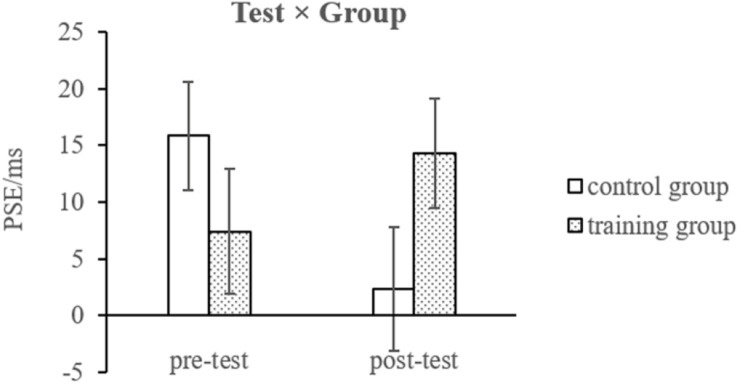
The interaction between test sessions and groups for points of subjective equality (PSEs). The error bars represent standard errors of the means.

**FIGURE 5:**
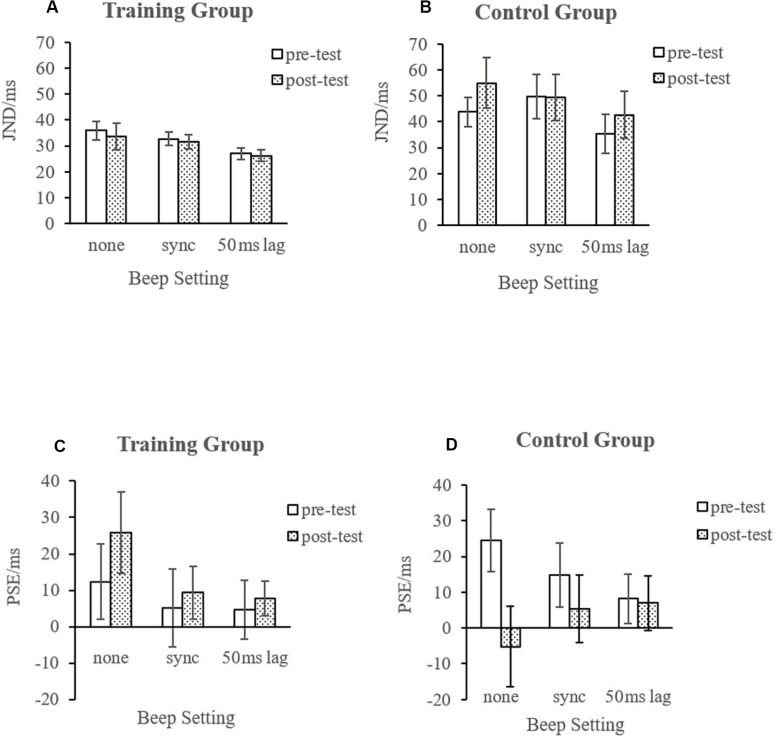
Average PSEs and just-noticeable differences (JNDs). The error bars represent standard errors of the means. (**A,C** for training group; **B,D** for control group).

For PSE (Figures 4, 5), the analysis revealed a significant three-way interaction [*F*(2, 46) = 5.833, *p* = 0.006, η^2^ = 0.202] and a significant interaction between test sessions and groups [*F*(1, 23) = 5.492, *p* = 0.028, η^2^ = 0.193]; no other effects were found.

Resolving the latter interaction by group showed opposite trends for the two groups from pre-test to post-test, namely, a non-significant shift of PSE to the right for the mindfulness group, *F*(1, 11) = 2.733, *p* = 0.127, and a likewise non-significant change of PSE to the left for the control group, *F*(1, 12) = 3.314, *p* = 0.094.

Resolving the three-way interaction by group revealed a non-significant beep setting × test session interaction for the mindfulness group, *F*(2, 22) = 1.366, *p* = 0.276, but significant for the control group, *F*(2, 24) = 4.794, *p* = 0.018. Importantly, in the mindfulness group, the effect of test session, with PSE shifting to the right, was significant in the visual-only condition only, *t*(11) = −2.561, *p* = 0.026, not in the two beep conditions, *t*(11) > −0.700, *p* > 0.5. In the control group, the effect of test session, with PSE shifting to the left, was likewise significant in the visual-only condition only, *t*(12) = 2.424, *p* = 0.032, not in the two beep conditions, *t*(12) < 1.100, *p* > 0.3. Further simple effect analysis indicated that only in the mindfulness group, in the post-test, the differences between the visual-only condition and synchronized condition (*p* = 0.058), and between the visual-only and 50 ms lag conditions (*p* = 0.070), have been magnified to some extent.

For JND (Figure 5), the main effect of the stimulus settings was significant [*F*(2, 46) = 6.932, *p* = 0.002, η^2^ = 0.232]. The JNDs were significantly smaller under the 50 ms lag condition (Figure 1C), indicating that a significant temporal ventriloquism effect only occurred under this condition. The results of the analysis of the PSE data indicated that after the 8-week training, participants had a larger bias, tending to perceive that the disks appeared more often from left to right. This bias was found only in the visual-only condition. However, when there were task-irrelevant beeps, this kind of bias disappeared.

Mean scores of the five factors of FFMQ for both groups are listed in Table 3. To further explore whether the differences in PSEs between the training group and the control group were related to participants’ mindfulness traits, multiple regressions were conducted (five factors of FFMQ, with group, test session, and beep setting as independent variables, and PSE as the dependent variable). The results revealed that the overall regression was significant [*F*(8, 135) = 2.872, *p* = 0.006], but among the individual traits, only the beta value of “acting with awareness” was significant [*t* = 3.822, *p* < 0.0001], while the other factors were not significant (“observing,” *t* = −0.026, *p* = 0.979; “describing,” *t* = 0.667, *p* = 0.506; “non-judging,” *t* = −0.623, *p* = 0.535; “non-reacting,” *t* = 1.106, *p* = 0.271; group, *t* = 1.253, *p* = 0.212; test session, *t* = −1.574, *p* = 0.118; beep setting, *t* = −1.085, *p* = 0.280).

**TABLE 3:** Mean Five Facet Mindfulness Questionnaire (FFMQ) scores with associated SEM for each group and both pre- and post-test.

		Observing	Describing	Acting with awareness	Non-judging	Non-reacting
Training	Pre	22.82 (1.01)	25.36 (1.03)	22.27 (1.00)	23.73 (1.38)	17.00 (0.84)
Group	Post	24.45 (1.49)	25.55 (1.08)	25.91 (1.07)	28.27 (1.26)	20.09 (0.61)
Control	Pre	21.31 (0.93)	25.15 (1.11)	23.62 (1.45)	22.38 (1.36)	17.92 (1.05)
Group	Post	21.15 (1.45)	25.54 (0.81)	23.85 (1.67)	25.62 (1.29)	10.00 (1.08)

## Discussion

The present study explored the effect of a relatively long (8-week) mindfulness training on a TOJ task and how it impacted TOJ under conditions of a temporal ventriloquism paradigm. Consistent with previous studies, a significant temporal ventriloquism effect was observed in both groups and across both test sessions, as the JNDs were reduced (i.e., sensitivity for TOJ improved) under the 50 ms lag condition. The most important finding of the present study lay in the significant interaction of the PSEs. The three-way interaction and the interaction between test sessions and groups indicated that for the visual-only condition, a left-to-right bias was dominant in participants who completed the 8-week mindfulness training in that they tended to judge that the left disk appeared first. However, under temporal ventriloquism conditions (for both synchronous and 50 ms lag settings), this induced bias disappeared. Moreover, the change in PSE was found to be correlated with the specific trait of “acting with awareness,” suggesting the role of a specific attentional factor in modulating time perception.

Multiple studies have confirmed that mindfulness training can enhance the activity of the attention system (Grossman et al., 2004; Jha et al., 2007; Semple, 2010). For example, Jha et al. (2007) investigated whether mindfulness training can modify or even enhance a specific subsystem of attention. Their findings indicated that naïve participants who took part in an 8-week MBSR (like that used in our study) demonstrated significant improvement in attentional orienting as compared to the control group (Jha et al., 2007). Tomasino and Fabbro (2016) have investigated the brain activation changes related to an 8-week mindfulness-oriented meditation training on an initially naïve subject cohort. They showed that meditation increased activation in the right dorsolateral prefrontal cortex (PFC) and the left caudate/anterior insula and decreased activation in the rostral PFC and right parietal area 3b, which is involved in sustaining and monitoring the focus of attention (Tomasino and Fabbro, 2016). Importantly, activation of the dorsolateral prefrontal cortex is also responsible for the temporal discrimination of sub-second intervals (Lewis and Miall, 2003a), and typically, the right hemispheric prefrontal cortex plays the predominant role in attentional processing during sub-second measurements (Lewis and Miall, 2006). We thus inferred that after mindfulness training, boosted activity in the dorsolateral PFC might contribute to the ability to discriminate the visual intervals between two disks (i.e., TOJ). Previous studies using temporal bisection tasks have found that the PSEs are altered after mindfulness-based training (Kramer et al., 2013; Droit-Volet et al., 2018; Singh and Srinivasan, 2019). To the best of our knowledge, the present study is the first to adopt an audiovisual TOJ paradigm to investigate the effects of mindfulness training on time perception. PSE changes in the present study revealed that after 8 weeks of mindfulness training, there was a significant bias to believe that the left disk was presented first. The TOJ task makes strong demands on the attention, especially when the SOA between the two consecutive visual stimuli is short. Indeed, the TOJ task requires deliberate attention. Performance of the TOJ task is probably associated with forming representations of stimuli as separate and temporally ordered sensory events, mobilizing a wide brain network including the prefrontal cortex, the parietal lobules (superior and inferior), and the occipitotemporal regions (Binder, 2015).

In this study, the two disks were presented on either the left or right locations on the screen. TOJ performance, therefore, required discrimination of the spatial location of the two disks. Previous studies have shown that attentional orienting triggers a prior-entry effect that modulates TOJ (Schneider and Bavelier, 2003; Schettino et al., 2016), in that the attentional focus on a given modality or a given spatial location helps the participants identify the first target in a TOJ task. Although we did not explicitly manipulate the attentional cues by orienting subjects’ attention to a particular part of the screen, we argue that after mindfulness training, the allocation of attention on subjective perception of the relative timing of target stimuli (two disks) was improved, i.e., with a shift to the internally oriented attention (Glicksohn, 2001), and this enhanced the “left-to-right” bias. As a result, the PSE became significantly larger post-test for the training group under visual-only conditions. In this way, the TOJ performance was consistent with the altered PSEs. With that said, one should be careful about drawing such conclusions pending more empirical experimental evidence that directly manipulates attentional cueing on the TOJ task. Furthermore, in present study, we used a sparse sampling with 50 ms as a step size for time intervals between two disks; this might reduce the sensitivities of TOJ, as compared with previous denser sampling (such as 12 ms step size in Morein-Zamir et al., 2003). This limitation should be addressed with further study.

Under the temporal ventriloquism conditions, the task-irrelevant beep probably played a role as a re-calibration in space (allocation) of attention when participants received cross-modal stimuli and thus facilitated the TOJ task while attenuating the left-to-right bias, as auditory stimuli tend to capture one’s attention automatically (Koelewijn et al., 2009). On the other hand, neuroimaging evidence has shown that greater temporal lobe activity can be observed during measurement of a briefer interval (compared with intervals longer than 1 s), suggesting the preferential use of auditory imagery for measurement of short durations (Lewis and Miall, 2003a). After mindfulness training, the overall attentional capacity remains unchanged (Trautwein et al., 2020), but significant improvements in selective attention have been observed (Chiesa et al., 2011). In the present experiment, the left-to-right bias – which is caused mainly by enhanced visual attentional processes – may be weakened under the audiovisual conditions, as a result of the fact that auditory attention tends to take precedence in temporal estimation tasks. An analogous finding was reported by Manly et al. (2005), in which they showed that attention has been shifted to the right when alertness declines (Manly et al., 2005). In our case, after mindfulness training, the attentional capacities (probably including enhanced alertness) will facilitate the opposite – a shift to the left space, which further contributes to the left-to-right bias.

However, the above conclusions must be considered carefully. One might argue that an orthogonal design that included both horizontal and vertical positions of visual stimuli would minimize the left-to-right bias and thereby produce more meaningful data. To further clarify the role of attention in this study, direct tests of participants’ attention functions should be implemented. For example, we need to implement more behavioral analysis of participants’ attention functions, particularly with a focus on specific subsystems such as orienting (Jha et al., 2007) as well as detailed protocols for examining the roles of temporal and spatial attention in the TOJ task.

In summary, this study provides the first empirical evidence to show how modulation of “higher-level” cognitive functions, including cognitive states, by long-term mindfulness training can affect the “lower-level” perceptual discrimination of sub-second timing. Observers who received a long period (8 weeks) of mindfulness training gained the benefits of enhanced attentional awareness, which contributed to biased timing behavior by magnifying the left-to-right bias in discriminating the temporal order of visual events. However, this transfer effect might be short-lived and was not present when attention was diverted by auditory events in the cross-modal temporal ventriloquism illusion.

## Data Availability Statement

All datasets generated for this study are included in the article/supplementary material, further inquiries can be directed to the corresponding authors.

## Ethics Statement

The studies involving human participants were reviewed and approved by the Academic Affairs Committee of the School of Psychological and Cognitive Sciences at the Peking University. The patients/participants provided their written informed consent to participate in this study.

## Author Contributions

XL and LC designed the experiments. YT performed the experiments and analyzed the data. YT, XL, and LC wrote the manuscript. All authors contributed to the article and approved the submitted version.

## Conflict of Interest

The authors declare that the research was conducted in the absence of any commercial or financial relationships that could be construed as a potential conflict of interest.
